# Investigation of the Impact of Antibiotic Administration on the Preterm Infants’ Gut Microbiome Using Next-Generation Sequencing—Based 16S rRNA Gene Analysis

**DOI:** 10.3390/antibiotics13100977

**Published:** 2024-10-16

**Authors:** Ahmet Aktaş, Berkay Yekta Ekren, Beril Yaşa, Osman Uğur Sezerman, Yaşar Nakipoğlu

**Affiliations:** 1 Medical Microbiology Department, Istanbul Faculty of Medicine, Istanbul University, 34093 Istanbul, Türkiye; yasarnakip@istanbul.edu.tr; 2 Department of Biostatistics and Bioinformatics, Acıbadem MAA University, 34752 Istanbul, Türkiye; berkayekren@gmail.com (B.Y.E.); ugur.sezerman@acibadem.edu.tr (O.U.S.); 3 Child Health and Diseases Department, Istanbul Faculty of Medicine, Istanbul University, 34093 Istanbul, Türkiye; beril.yasa@istanbul.edu.tr

**Keywords:** microbiota, preterm infants, antibiotic, vancomycin-resistant *Enterococcus*, carbapenem-resistant *Klebsiella pneumoniae*, 16S rRNA, meconium, microbiome

## Abstract

**Background:** The human gut microbiota is an extensive population of microorganisms, and it shows significant variations between periods of optimal health and periods of illness. Vancomycin-resistant *Enterococcus* (VRE) and carbapenem-resistant *Klebsiella pneumoniae* (CRKP) are both pathogenic agents (BPAs) that can colonize in the gut after dysbiosis of microbiotal composition following antibiotic treatment. **Methods:** This study aimed to investigate the impact of antibiotics on the microbiotal composition of the gut. For this purpose, the first pass meconiums of 20 patients and the first rectal swabs containing BPAs of the same patients after antibiotic treatment were studied using next-generation sequencing-based 16S rRNA gene analysis. The V1–V9 region of 16S rRNA was sequenced with Oxford Nanopore. **Results:** Twenty-five phyla were detected in the meconiums, and 12 of them were absent after antibiotic treatment. The four most prevalent phyla in meconiums were Bacillota, Pseudomonadota, Bacteroidota, and Actinomycetota. Only the relative abundance of Pseudomonadota was increased, while a significant decrease was observed in the other three phyla (*p* < 0.05). A significant decrease was observed in alpha-diversity in rectal swabs containing BPAs versus meconiums (*p* = 0.00408), whereas an increased variance was observed in beta-diversity in all samples (*p* < 0.05). As a result of a LEfSe analysis, Pseudomonadota was found to have a higher relative abundance in rectal swabs, and Bacillota was significantly higher in the meconiums of the twins. **Conclusions:** Our study strongly verified the relationship between the administration of antibiotics, dysbiosis, and colonization of BPAs in the infants’ gut microbiota. Further research would be beneficial and needed, comprising the natural development process of the infants’ gut microbiota.

## 1. Introduction

The microbiota consists of around 10^14^ symbiotic microbial cells that have adapted to inhabit the gut [1]. The gut becomes colonized with facultative anaerobic bacteria following birth, due to a partially aerobic or microaerophilic environment. These bacteria create an environment conducive to anaerobic bacteria by utilizing the oxygen present in the environment [2]. As a result, with the introduction of nutrition (breast milk or formula), this process undergoes a transformation, allowing for the proliferation of *Bifidobacterium* spp., *Bacteroides* spp., and *Clostridium* spp. This process facilitates the development of facultative anaerobic bacteria. Initially, the population of Pseudomonadota and *Enterobacteriaceae* undergoes a decline, while that of Bacteroidota and *Bifidobacterium* spp. increases. Over a period of two to four years, the infants’ gut microbiota undergoes a transformation into its adult form. This process commences with the establishment of the Bacillota and Bacteroidota, which are subsequently succeeded by the Pseudomonadota, Actinomycetota, and Verrucomicrobiota [2,3,4]. These bacteria play crucial roles in human physiology, such as completing the digestion of foods and altering specific vitamins [5].

Approximately 160 species, known as the core microbiota, are present in every individual, despite a healthy humans’ gut microbiota consisting of more than 1000 species [6,7]. Under normal circumstances, a robust gut microbiota prevents the establishment of harmful microorganisms [8]. But when the distribution of gut microbiota composition is disturbed, which is called dysbiosis, mostly as a result of the administration of antibiotics and other factors such as the duration of pregnancy, the birth method, and use of breast milk, then pathogenic bacteria colonize in the gut [9,10,11,12,13]. To study the gut microbiota using conventional techniques such as stool samples or rectal swab cultures, the isolation, identification, and enumeration of bacteria is difficult and time-consuming, and we were able to isolate only 10–25% of the microbiota. The majority of gut bacteria are anaerobic, in addition to diversity in the species and numbers of bacteria [4].

Next-generation sequencing techniques have made it possible to study the variety of microorganisms in the gut of neonates by analyzing the 16S rRNA sequence [14]. The 16S rRNA molecule is about 1500 base pairs long and has nine areas where the sequence changes significantly. Utilizing primers that target several variable areas enables the attainment of diverse levels of accuracy and specificity in the obtained outcomes [15,16].

Vancomycin-resistant *Enterococcus* (VRE) and carbapenem-resistant *Klebsiella pneumoniae* (CRKP) are two major causes of different nosocomial infections such as urinary tract, blood stream, septicemia, and others. VRE and CRKP are carried on unhygienic health worker hands and colonized in the gut microbiota of hospitalized patients suffering from dysbiosis. The aim of this current study is to find out the impact of antibiotics in the dysbiosis of the gut microbiota of preterm infants and to calculate the colonization time for VRE and CRKP.

## 2. Results

### 2.1. Evaluation of Infants’ Microbiota

#### 2.1.1. Microbial Diversty Analysis

**Figure 1:**
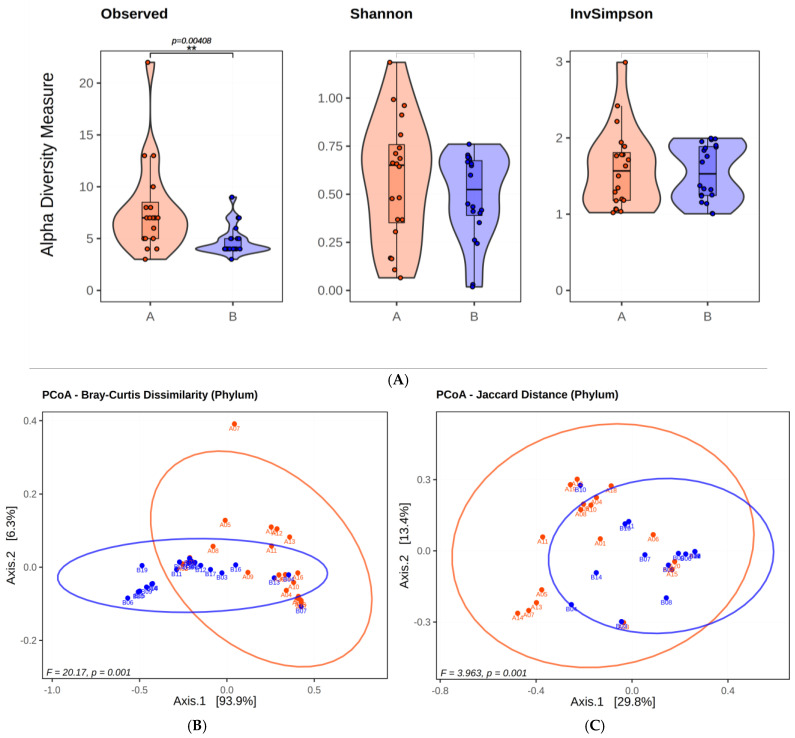
The results of the alpha and beta diversity calculations at the phylum level are presented in a visual format. The observed, Shannon, and InvSimpson calculations are displayed in the (**A**) alpha diversity visualization. The presentation employs a combination of violin and box plots, with the middle thick line in the box plot representing the median. In (**B**,**C**), Jaccard and Bray–Curtis dissimilarity distances, which are beta diversity metrics, are presented on the PCoA graph. The ellipses were drawn using the 95% confidence interval. In (**A**–**C**) the blue color indicates meconiums, and the red color indicates BPAs.

Observed alpha-diversity showed a statistically significant difference between the groups (meconiums vs. BPAs) (*p* = 0.00408). However, the other two metrics that consider quantities, the Shannon and Inverse Simpson (InvSimpson) indices, did not show statistically significant differences (*p* = 0.39 and 0.90, respectively). In beta-diversity analysis, although Jaccard distance (F = 3.963, *p* = 0.001) and Bray–Curtis difference (F = 20.17, *p* = 0.001) did not separate the groups, an increase in variance was observed (Figure 1) (Supplement Files S1 and S2).

#### 2.1.2. Analysis of Infants’ Microbiota at Phyla Level

**Figure 2:**
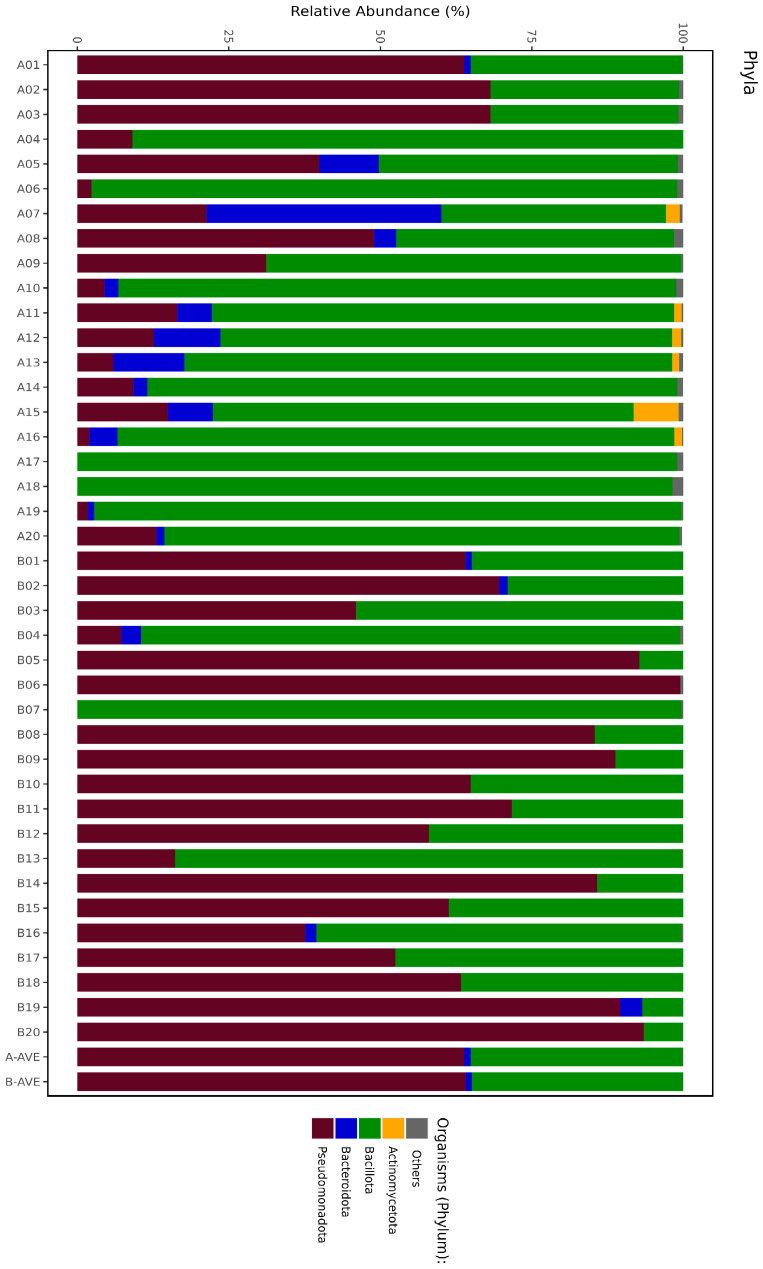
The stacked graphs of phyla. In the figure, “A” represents meconium and “B” represents BPAs. (The chart classifies branches below 1% as others. For interpretation of the references to color in this figure legend, the reader is referred to Supplement File S3).

All infants were administered gentamicin (GEN) and ampicillin (AMP) (GAMP) and other antibiotics during hospitalization (Supplement File S1). Meconium samples revealed 25 phyla versus 13 in rectal swabs after antibiotic treatment, which obviously showed that 12 phyla were absent after antibiotic treatment (Figure 2).

Table 1 shows 25 phyla of meconium (A) and 13 phyla of BPAs after antibiotic treatment (B). To interpret the impact of administered antibiotics on gut microbial composition, 25 phyla of meconium samples were divided into three groups according to their sensitivities to antibiotics and pathogenicity in humans:

- 1st Group. Seven phyla were within the antibiotic spectrum and known to cause human disease.
- 2nd Group. Seven phyla were within the antibiotic spectrum but did not cause human disease.
- 3rd Group. Eleven phyla were not causing human disease nor in the spectrum of antibiotics.

**Table 1:** Phyla Detected in Meconiums (A) and BPAs (B).

Phyla	Number of Samples		Average Incidence Percentage (%)		*p*-Value (<0.05)	Comment
	A	B	A	B		
**1st Group: Phyla related with human disease and within antibiotic efficiency spectrum**						
Bacillota	20	20	71.851	36.9886	0.0009319	**Significant decrease**
Pseudomonadota	20	20	21.7138	62.3941	0.00006	**Significant increase**
Bacteroidota	19	17	5.157	0.565	0.006066	**Significant decrease**
Actinomycetota	19	19	1.0071	0.0423	0.00005	**Significant decrease**
Fusobacteriota	4	1	0.0285	0.00001	0.1056454	not-significant
Campylobacterota	5	4	0.0067	0.0041	0.3525421	not-significant
Spirochaetota	5	1	0.0033	0.0001	0.0590582	not-significant
**2nd Group: Phyla not related with human disease but within antibiotic efficiency spectrum**						
Cyanobacteriota	13	3	0.0655	0.0005	0.001511	**Significant decrease**
Verrucomicrobiota	7	3	0.0246	0.0006	0.0243902	**Significant decrease**
Myxococcota	2	0	0.019	0	0.3710934	not-significant
Deinococcota	4	0	0.0053	0	0.1003482	not-significant
Chloroflexota	2	2	0.0009	0.0004	0.4226781	not-significant
Gemmatimonadota	1	0	0.0002	0	1	not-significant
Aquificota	3	0	0.0042	0	0.1814492	not-significant
**3rd Group: Phyla not related with human disease and not within antibiotic efficiency spectrum**						
Thermotogota	5	0	0.0402	0	0.0590582	not-significant
Thermodesulfobacteriota	9	3	0.0277	0.0034	0.0366713	**Significant decrease**
Candidatus Melainabacteria	7	0	0.0173	0	0.0222541	**Significant decrease**
Thermomicrobiota	1	0	0.0041	0	1	not-significant
Bdellovibrionota	2	0	0.0021	0	0.3710934	not-significant
Chrysiogenota	2	0	0.0008	0	0.3710934	not-significant
Rhodothermota	2	1	0.0004	0.0002	1	not-significant
Nitrospirota	1	1	0.0003	0	1	not-significant
Candidatus Thermoplasmatota	1	0	0.0002	0	1	not-significant
Nitrospinota	1	0	0.0002	0	1	not-significant
Lentisphaerota	1	2	0.0001	0.0005	0.5862137	not-significant

Fourteen of the meconium phyla (phyla in the 1st and 2nd groups) were in the efficacy spectrum of administered antibiotics, and 11 (phyla in group 3) were out of spectrum. We detected that all (except Pseudomonadota) five phyla (Bacillota, Bacteroidota, Actinomycetota, Cyanobacteriota, and Verrucomicrobiota) were significantly decreased in number or completely absent in rectal swabs after antibiotic treatment (*p* < 0.05). Two other phyla (Thermodesulfobacteriota, Candidatus Melainabacteria) in the 3rd group were also absent, more likely due to negative interaction with other phyla after antibiotic treatment (Supplement File S3).

#### 2.1.3. Microbial Diversity Analysis at Species Level

A total of 2594 species were identified in meconiums and BPAs. 924 (35.6%) were detected only in meconiums and 574 (22.1%) only in BPAs after antibiotic treatment, and the remaining 1096 species existed in both samples. Significant differences were found in 385 species (*p* < 0.05). The first three species with the highest relative abundance were identified as *Enterococcus* spp., *Klebsiella* spp., and *Staphylococcus* spp. in rectal swabs containing BPAs. *Klebsiella* spp. and *Enterococcus* spp. were the first two most common species, while *Klebsiella* spp. and *Staphylococcus* spp. were the two most common species detected in meconiums. Despite an increase in *Bifidobacterium* spp. percentage in BPAs (0.004% to 0.0104%), we determined that this increase was not significant (*p* > 0.05). *Akkermansia* spp. and *Blautia* spp. showed a significant decrease in rectal swabs containing BPAs comparing with meconium samples from 0.0236% to 0.0006% (*p* = 0.04232) and from 6.8737% to 1.0573% (*p* = 0.000514), respectively (Supplement File S4).

**Figure 3:**
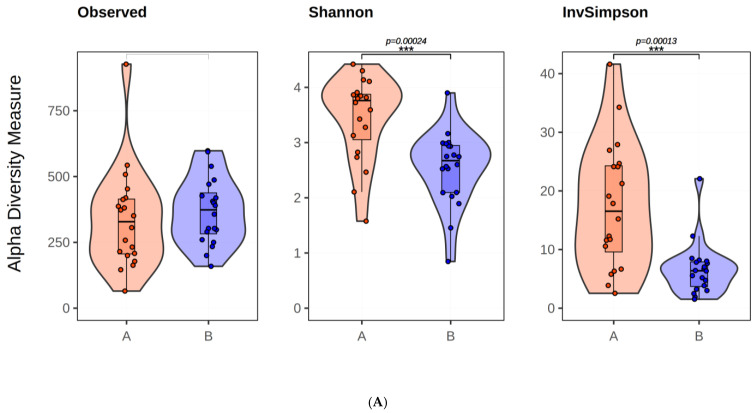

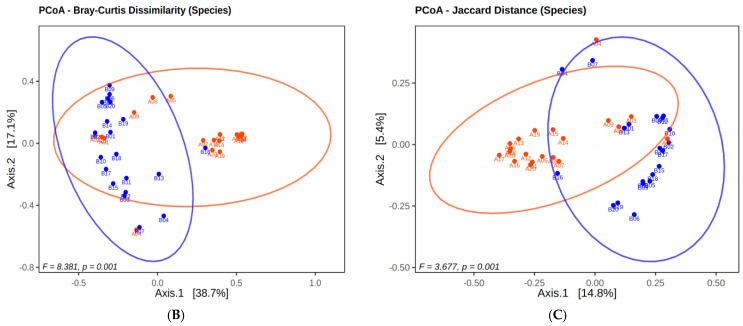
The results obtained in the alpha-diversity (**A**), Bray–Curtis (**B**), and Jaccard (**C**) PCoA graph. In (**A**–**C**) the blue color indicates meconiums, and the red color indicates BPAs.

While the observed alpha-diversity measurements showed no significant difference (*p* > 0.05), the Shannon (*p* = 0.00024) and InvSimpson (*p* = 0.00013) measurements showed significant decreases. The difference can be attributed to different amounts of bacteria. This indicates a change in the amount of the same bacteria in the two sample groups. Regarding the beta diversity analysis, the PCoA plot for both Jaccard and Bray–Curtis dissimilarity distances shows the colonization of different species between the two groups (Figure 3) (Supplement Files S5 and S6). Using the UPGMA method to construct a phylogenetic tree based on the distance data calculated for beta diversity, we found that Jaccard distances separated groups more effectively than Bray–Curtis (F = 8.381, *p* = 0.001). The Jaccard distance matrix (F = 3.677, *p* = 0.001) divided the samples in this tree into two clusters, with 3 B and 16 A group samples in one cluster and 17 B and 4 A group samples in the second cluster.

### 2.2. Evaluation of Twin Infants

#### 2.2.1. Microbial Diversity Analysis in Twins

**Figure 4:**
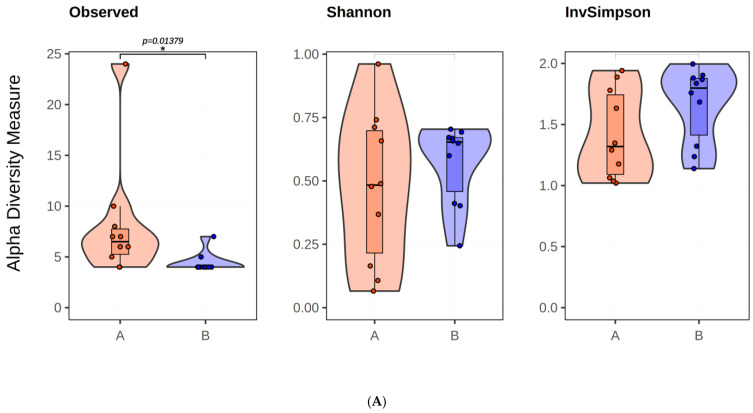

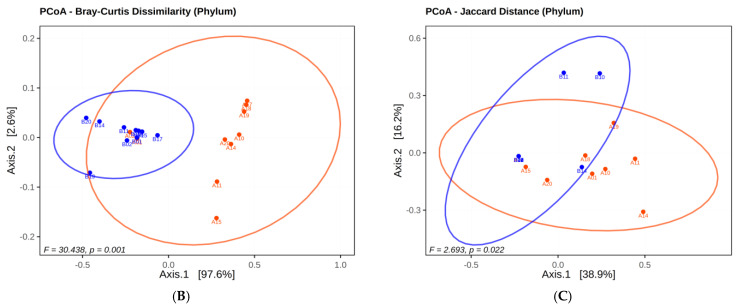
Alpha diversity plots (**A**), PCoA plots of Bray–Curtis dissimilarity (**B**) and Jaccard distance (**C**). In images (**A**–**C**), the blue color indicates meconiums, and the red color indicates rectal swabs containing BPAs.

A significant difference (*p* = 0.0075) was shown in alpha diversity, while Shannon and InvSimpson metrics did not show statistically significant differences (*p* = 0.3886 and *p* = 0.1654, respectively). In beta-diversity analysis, Jaccard distance (F = 30.438, *p* = 0.001) and Bray–Curtis difference (F = 2.693, *p* = 0.022) revealed reduced variance, although not significant, for meconium and in rectal swabs containing BPAs (Figure 4).

#### 2.2.2. Analysis of Twin Infants’ Microbiota at Phyla Level

A total of 10 out of 20 infants were twins (infants no. 1–2, 10–11, 14–15, 17–18, and 19–20). Colonization of VRE and CRKP in the gut is considered an indicator of dysbiosis. All infants were delivered via cesarean section and breastfed during their hospitalization. The main factor that might play an important role in dysbiosis is antibiotics. Three phyla (Cyanobacteriota, Verrucomicrobiota, and Aquificota) were absent in infant no. 1 after treatment with teicoplanin and cefotaxime for four days. All three phyla do not cause disease in humans (2nd group in Table 1). Four phyla (Actinomycetota, Pseudomonadota, Bacillota, and Bacteroidota) were detected in twins no. 1 and 2 after antibiotic treatment, and the colonized time for VRE and CRKP was 25 days in each.

There were 7 and 10 phyla in meconium samples of twins 10 and 11, respectively. Rectal samples of twins after antibiotic treatment showed absences of 3 out of 7 phyla (Bacteroidota, Candidatus Melainabacteria, and Verrucomicrobiota) in twin no. 10 and 6 out of 10 phyla in no. 11 (Cyanobacteriota, Bacteroidota, Candidatus Melainabacteria, Verrucomicrobiota, Spirochaetota, and Deinococcota) when treated with antibiotics. The colonization time in infant no. 10 was 10 days earlier than in infant no. 11. Although vancomycin and teicoplanin are both classified as glycopeptide antibiotic groups, it seems that use of vancomycin in infant no. 10 was more effective on microbiota composition than teicoplanin. On the other hand, although the same antibiotic protocol was administered in twins 14 and 15, dysbiosis took place 14 days later in infant no. 14 than in infant no. 15. The rich and diverse microbiota composition of meconium samples in infant no. 14 (22 phyla) compared with infant no. 15 (6 phyla) obviously reflect the antibiotic efficacy results being seven and four phyla in twins no. 14 and 15, respectively. We found the same result in twins no. 17 and 18 as in twins no. 14 and 15. The colonization time in infant no. 17 was eight days later than in infant no. 18, probably due to the richness of the microbiota composition of meconium samples in infant no. 18 (6 phyla) compared with infant no. 15 (4 phyla). In twins 19 and 20, the same antibiotic protocol was applied in each, and the same four phyla (Actinomycetota, Pseudomonadota, Bacillota, and Bacteroidota) existed in each twin after antibiotic administration, so the colonization time for infant no. 19 was very close (29 days) to infant no. 20 (30 days) (Supplement File S1).

#### 2.2.3. LEfSe Analysis Results

**Figure 5:**
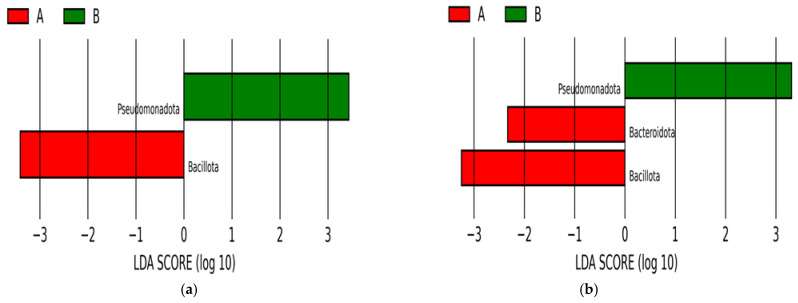
LEfSe at Phylum level. LEfSe analysis of the meconiums (A) vs. BPAs (B) at phylum level. While the red color represents bacteria found in meconiums, green represents the phyla in BPAs, separating the groups. As the image (**a**) represents the analysis performed on only the twins’ samples, (**b**) shows the LEfSe conducted for all of the samples.

When the LEfSe analysis was carried out for twins at phylum level, two identifyr phyla were determined to be most effective, Pseudomonadota and Bacillota. As Pseudomonadota was found with a higher relative abundance in meconiums, Bacillota was significantly higher in the meconiums of the twins. However, when all of the samples were included in the analysis, the phylum Bacteroidota was found effective and present mostly in meconiums, in addition to the other two phyla belonging to the bacterial domain (Figure 5).

**Figure 6:**
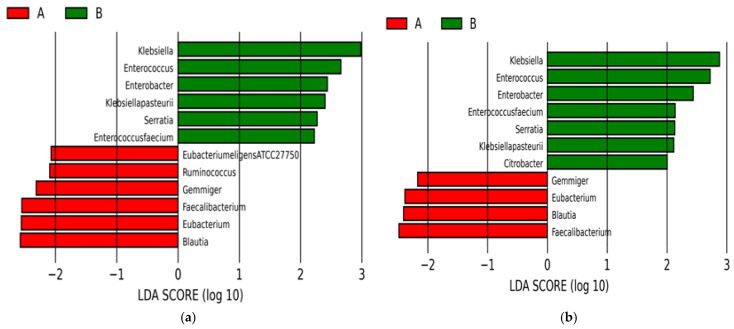
LEfSe analyses of twins’ (A) and all (B) samples at species-level. While the red color represents species-level bacteria found in meconiums, green represents the species in BPAs discriminating between the meconiums and BPAs. As the image (**a**) represents the analysis performed on only the twins’ samples, (**b**) shows the LEfSe conducted for all of the samples.

As a result of species-level analysis, 12 bacteria were found to be important: *Klebsiella* spp., *Enterococcus* spp., *Enterobacter* spp., *Klebsiella pasteurii*, *Serratia* spp., *Enterococcus faecium* more enriched in BPAs and members of Pseudomonadota, and *Eubacterium eligens* ATCC 27750, *Ruminococcus* spp., *Gemmiger* spp., *Faecalibacterium* spp., *Eubacterium* spp., and *Blautia* spp. were found to be important in separating the groups of twins’ data. On the other hand, only 11 species were found to be highly effective in the separation between the meconiums and BPAs in the whole data. As a more enriched species of the BPAs, *Citrobacter* spp. was the only addition. Only *Faecalibacterium* spp., *Blautia* spp., *Eubacterium* spp., and *Gemmiger* spp. of the Bacillota phylum were found to be significant as the result of the analysis (Figure 6).

## 3. Discussion

In recent years, studies have been conducted to investigate the potential relationship between the human intestinal microbiota and human health. In 2019, Jethwani and Grover [17] found that the most common phyla in the gut microbiota of healthy people are Bacteroidota, Bacillota, Pseudomonadota, Fusobacteriota, Mycoplasmatota, Actinomycetota, and Verrucomicrobiota. These phyla make up 90% of the total microbiota. In the Bäckhed [18] study, Bacillota and Bacteroidota were most prevalent in rectal samples of 1-year-old Swedish babies, followed by Actinomycetota and Pseudomonadota. Despite the similarities between the 25 phyla we identified in meconium and those found in current studies, we detected differences in the rankings. In our study, the phyla of bacteria according to their relative abundance was determined as follows: Bacillota, Pseudomonadota, Bacteroidota, Actinomycetota, Cyanobacteriota, Thermotogota, Fusobacteriota, Thermodesulfobacteriota, and Verrucomicrobiota. This discrepancy may be attributed to the use of low-weight infants’ meconium instead of adult rectal samples in our study. The mode of delivery influences the infants’ gut microbiota. In infants born vaginally, the dominant bacterial genera are *Lactobacillus* spp. and *Prevotella* spp. On the other hand, the most common genera in infants born by cesarean section are those typically found on the skin, such as *Streptococcus* spp., *Cutibacterium* spp., *Corynebacterium* spp., and *Staphylococcus* spp. This shift is accompanied by a decrease in the abundance of *Bifidobacterium* spp. and *Bacteroides* spp. [2].

Broad-spectrum antibiotics reduce the diversity of the gut microbiota and cause dysbiosis by destroying beneficial bacteria as well as killing pathogens [19]. The use of antibiotics for prophylaxis and treatment disrupts the balance and diversity of the gut microbiota in hospitalized infants. Healthcare workers who disregard antisepsis rules may harbor multidrug-resistant pathogens like VRE and CRKP, which can colonize infants’ guts under these conditions. These pathogens can disseminate rapidly in environments with compromised microbiota, such as preterm infants, resulting in mortality. Studies have demonstrated that these infections are responsible for 35% of preterm infant mortality [20,21]. Therefore, most studies on nosocomial infection have focused on the prevalence or treatment of these two pathogens [22,23].

GAMP administration impacts the gut microbiotas’ biodiversity. Gasparini et al. [24] found that GAMP administration increased the relative abundance of *Enterococcus* spp. while decreasing the relative abundance of Bacteroidota and *Bifidobacterium* spp. In contrast, Fouhy et al. [25] observed an increase in the relative abundance of Pseudomonadota and a decrease in the relative abundance of *Lactobacillus* spp. and *Bifidobacterium* spp. with GAMP administration. Similar to these data, in our study, we also detected a significant increase in the relative abundance of Pseudomonadota and a decrease in the relative abundance of Bacteroidota. However, we detected an increase in the relative abundance of *Bifidobacterium* spp., which was not significant (*p* = 1). We attribute the rise in Pseudomonatoda to the high percentage of enteric bacteria within this phylum instead of Bacillota, and prolonged hospitalization (25–96 days) of these infants might facilitate the growth of this phylum.

Dardas et al. [26] observed an increase in the relative abundance of Bacteroidota following GAMP administration that persisted for up to 30 days. Furthermore, Zwittink et al. [27] found that using amoxicillin-GEN for more than five days led to an increase in the relative abundance of Bacteroidota and a decrease in the relative abundance of *Bifidobacterium* spp. In contrast to these findings, our study revealed a significant decrease in the relative abundance of Bacteroidota.

Pérez-Cobas et al. [28] reported that bacteriostatic antibiotics increase the expression of genes that contribute to the synthesis of the liposaccharide layer in Gram-negative bacteria. They also demonstrated that bactericidal antibiotics increase the number of genes involved in the formation of the endospore, resulting in an increase in the number of Gram-positive bacteria. In our study, when assessing *Prevotella* spp., *Bacteroides* spp., *Porphyromonas* spp., *Flavobacterium* spp., *Nocardia* spp., and *Actinomycetes* spp. in BPAs, compared to meconiums, neither in twins (*p* = 0.5233, *p* = 0.343, *p* = 1, *p* = 1, *p* = 1, *p* = 1, respectively) nor in non-twin infants (*p* = 0.5938, *p* = 0.2366, *p* = 1, *p* = 1, *p* = 1, *p* = 1, respectively), no significant difference was observed.

*Bifidobacterium* spp. produces acetate and lactate through fermentation. Furthermore, it contributes to physiological development by facilitating the digestion of sugars present in breast milk that are not readily digestible by the infant. During breastfeeding, the number of these organisms in the gut increases, and they become among the dominant bacterial taxa. Wandro et al. [29] demonstrated that *Bifidobacterium* spp. was completely eliminated from the preterm infants’ gut microbiota after undergoing GAMP administration. The infants included in the study were fed breast milk during their stay in the ward. Despite the administration of vitamin K supplementation, no discernible increase in the relative abundance of *Bifidobacterium* spp. was observed in the infants. Another species, *Eggerthella* spp., also affects physiological development [30]. It contributes to the anticarcinogenic mechanism by fermenting sugars and synthesizing s-equol, which stimulates natural killer (NK) cells. In our study, we observed a significant decrease in the relative abundance of *Eggrethella* spp. in BPAs (*p* = 0.00909).

The data on the effects of gentamicin and beta-lactams on the preterm gut microbiota exhibit inconsistencies. In preterm infants treated with beta-lactam antibiotics, Lu et al. [31] found a significant decrease in the relative abundance of Bacteroidota and a significant increase in *Enterococcus* spp. Korpela et al. [32] examined the infants’ meconium microbiota. They found that gentamicin against *Enterococcus* spp. affected *Bifidobacterium* spp., resulting in less alpha-diversity. Drell et al. [33] demonstrated that GAMP administration resulted in a reduction in alpha-diversity scores in 50 preterm infants with birth weights below 1200 g. They observed that this treatment led to an increase in the relative abundance of *Staphylococcus* spp. and *Enterobacteriaceae*. Furthermore, the number of probiotic species, including *Bifidobacterium* spp. and *Lactobacillus* spp., also decreased. The results of our study demonstrated a significant decrease in the relative abundance of Bacillota and Bacteroidota, while Pseudomonadota was dominant, in accordance with previous studies, and a significant decrease in alpha diversity was observed at both the phylum and species levels. Additionally, a significant decrease in the relative abundance of *Lactobacillus* spp. (*p* = 0.00048) and an insignificant increase in the relative abundance of *Bifidobacterium* spp. (0.004% to 0.0104%, *p* = 1) were observed. Although not significant, a decrease in the relative abundance of *Staphylococcus* spp. was observed (1.7842% to 0.3446%, *p* = 0.92).

## 4. Material and Methods

### 4.1. Determination of Patient Population and Isolation of VRE and CRKP from Rectal Samples

To follow up on the gut microbiota composition in rectal samples after antibiotic usage in preterm infants, 20 preterm infants who were hospitalized at the neonatal intensive care unit (NICU) between January 2022 and August 2023 in Istanbul Medical Faculty, born before 32 weeks of age and weighing less than 1500 g, were included in the study (Table 2). All infants included in the study were born by cesarean section. The infants were fed with mothers’ milk via nasogastric tube, and breastmilk of the mother was applied through the nasal cavity as a unit protocol. Skin-to-skin contact was performed for all infants through the second week of life. Mothers were not screened for GBS prior to delivery and cefazolin was administered to mothers just before surgery, up to 30 min before delivery, as a surgical prophylaxis. Infants born to mothers with chorioamnionitis or a history of antibiotic use during pregnancy and those who received antibiotics before the first meconium passage were excluded from the study.

All preterm infants within the first 24 h of meconium sample cultures (n = 20, 2 g of each) that revealed growth and non-growth of vancomycin susceptible *Enterococcus* (VSE) and carbapenem susceptible *K. pneumoniae* (CSKP) by conventional methods were included in the study. Rectal samples were taken from the same infants every week until the isolation of both VRE and CRKP, using bile esculin agar supplemented with 6 mg/L vancomycin and MacConkey agar supplemented with 1 mg/L meropenem, respectively [34]. Susceptibilities to antibiotics for both agents were performed and confirmed by using conventional methods, as advised by the European Committee on Antimicrobial Susceptibility Testing (EUCAST) [35]. Rectal samples were taken from the newborns weekly during their stay in the NICU, and they were screened for VRE and CRKP. Meconiums (n = 20) and the first rectal samples containing VRE and CRKP (BPAs) (n = 20) were kept at −80 °C until gDNA isolation.

### 4.2. gDNA Extraction and 16S rRNA Sequencing

The gDNA was extracted using the Zymo BIOMICS DNA Miniprep (Zymo Research, Tustin, CA, USA, Cat. No. D4300) according to the manufacturers’ instructions. The 3rd generation sequencing technology, Oxford Nanopore (ONT, Oxford, UK), was utilized as the sequencing platform to specifically target the whole 16S rRNA region, which consists of polymerase chain reaction (PCR) amplicons that are around 1500 base pairs in length. The purity of the 16S amplicons was assessed using NanoDrop (Thermo Fisher Scientific, Wilmington, DE, USA). The reference ONT purity values for the 260/280 ratio ranging from 1.70 to 1.90, and the 260/230 ratio from 2.00 to 2.20, were determined to be the acceptable ranges for DNA extraction.

In order to prepare the amplicons for sequencing, the ONT kit, SQK-16S024, was utilized along with the instructions and primers 27F (5′-AGAGTTTGATCCTGGCTCAG-3′) and 1492R (5′-GGTTACCTTGTTACGACTT-3′) for 16S PCR amplification of all 16S regions [36]. The concentrations of the PCR products were assessed using Qubit 2.0 (ThermoFisher Scientific, Paisley, UK). Subsequently, the PCR products were combined to create the 16S DNA amplicon library. The produced library was combined with the ONT sequencing buffer, loading beads, and the required volume of DNase/RNase-free water. The resulting mixture was then loaded into an ONT FLO-MIN106D (version R9.4.1) flow cell. The MinION Mk1B (v. 23.07.12) was utilized to perform sequencing of the 16S library for each sample.

### 4.3. Bioinformatics and Statistical Analysis

In total, 1,418,944 reads and 1.4 Gbp bases were obtained. The N50 value was 1442 kb, and the average number of reads per sample was 34,608. The raw data were recovered from adapters and barcodes using the ONT Guppy (v. 6.1.5) command-line program. The quality control of reads thereafter consensus sequences were generated using the bbtools (v. 38.91), samtools (v. 1.13), and magicblast (v. 1.6). The resulting consensus reads were mapped to the NCBI 16S database (21 December 2023) and annotated to generate amplicon sequence variants (ASVs) tables for each sample.

The normality of the data was analyzed using the Shapiro–Wilk test, and differences between groups were determined by applying Welchs’ *t*-test to normally distributed samples and the Wilcoxon signed-rank test to non-normally distributed samples. The beta-diversity difference between meconiums and BPAs was evaluated by the permanova test. A higher F value indicates better grouping.

The ASV files were merged into a single table, and the relative abundance of bacteria was listed using R (v. 4.0.4) and Rstudio IDE (v. 1.4). The phyloseq package in R was employed to calculate alpha diversity metrics. Prior to diversity calculations, rarefaction was performed to an appropriate depth. Three metrics were employed to analyze alpha-diversity. First, observed features were used to explain the richness only. Subsequently, the Shannon Diversity Index was employed to elucidate both richness and evenness, with equal weight assigned to both. Finally, the Inverse Simpson (InvSimpson) Index was utilized to elucidate both richness and evenness, with greater emphasis placed on evenness. Beta diversity analysis was conducted using both the Jaccard and Bray–Curtis dissimilarity distances. The Jaccard metric determines the presence or absence of an organism at the analyzed taxonomic level (phylum, and species), whereas the Bray–Curtis metric also takes into account the number of organisms. In this study, the statistical significance was set at *p* < 0.05.

LEfSe analysis was conducted using the bioconda LEfSe (v. 1.1.2) tool, which is provided at https://github.com/SegataLab/lefse (access date: 11 October 2024) [37]. This analysis was carried out for all of the samples (20 samples in meconiums and 20 in BPAs) and twins (10 samples in meconiums and 10 in BPAs) separately. The created ASV files at different taxonomic levels (phylum, and species) were imported into the LEfSe software. With rarefaction, features with low abundance were filtered out. Then, a Wilcoxon-ranked test was carried out to identify the significant differences in relative abundance values between two groups. Features found to be significant were further analyzed to identify their effectiveness in separating the groups from each other using LDA (linear discriminant analysis). The LDA threshold was determined to be 2.0 for identification of most discriminative features between meconiums and BPAs. The bar plot visualizations were performed using the Python 3.12 matplotlib (v. 3.9.2) package.

## 5. Conclusions

In this study, Bacillota, Bacteroidota, Pseudomonadota, Actinomycetota, Verrucomicrobiota, *Bifidobacteriaceae*, and *Enterobacteriaceae* were found in the meconium microbiota, as well as *Cutibacterium* spp., *Corynebacterium* spp., and *Staphylococcus* spp. The presence of maternal skin bacteria such as Streptococcus spp. has been determined to be compatible with studies on this subject. However, the microbiota composition in BPAs showed a significant change, with a significant decrease in Bacteroidota, *Cutibacterium* spp., *Corynebacterium* spp., and *Streptococcus* spp., and a significant increase in Pseudomonadota and *Enterobacteriaceae*. This change may be due to the natural developmental process of the gut microbiota, cross-contamination by NICU staff, and antibiotics used during the NICU hospitalization of infants. Further studies at different age stages are needed to better understand the effect of antibiotics on human gut microbiota.

## Figures and Tables

**Table 2:** Birth information of the infants and the antibiotics they received during their stay in the intensive care unit.

Infant No **	Birth Age (Weeks + Days)	Birth Weight (g)	Antibiotics Used in Treatment * (Days)	Colonization of the First Mixed VRE + CRKP (Days)
**1**	**31 + 1/7**	**1470**	**GEN + AMP (3), TEC + CTX (4)**	**25**
**2**	**31 + 1/7**	**1410**	**GEN + AMP (3), TEC + CTX (4)**	**25**
3	29 + 2/7	855	GEN + AMP (3), **TEC + CTX (4)**	69
4	25 + 2/7	450	GEN + AMP (3), TEC + CTX (2), V + MEM (7), V + MEM + AK (9), LEV + SXT (7)	96
5	31 + 4/7	1385	GEN + AMP (3), TEC + CTX (4)	26
6	29 + 3/7	1200	GEN + P (3), TEC + GEN (6)	15
7	28 + 6/7	1440	GEN + SAM (3), TEC + TZP (4)	22
8	27 + 5/7	920	AMP + CTX (4), V + TZP (5)	40
9	27 + 4/7	640	GEN + AMP (3), TEC + CTX (4)	32
**10**	**29 + 4/7**	**1045**	**GEN + AMP (3), TEC + CTX (4), V + MEM (5)**	**35**
**11**	**29 + 4/7**	**1355**	**GEN + AMP (3), TEC + CTX (4), TEC + MEM (5)**	**45**
12	31 + 1/7	1270	GEN + AMP (3), TEC + CTX (3)	55
13	31	1425	GEN + AMP (4), TEC + CTX (3), V + TZP (3)	36
**14**	**29 + 5/7**	**1070**	**GEN + AMP (3), TEC + TZP (2)**	**74**
**15**	**29 + 5/7**	**1005**	**GEN + AMP (3), TEC + TZP (2)**	**60**
16	26 + 5/7	840	GEN + AMP (3), TEC + CTX (3)	33
**17**	**26 + 1/7**	**920**	**TEC + CTX (2), V + MEM (4)**	**60**
**18**	**26 + 1/7**	**930**	**TEC + CTX (2), V + MEM (4)**	**68**
**19**	**29 + 3/7**	**1350**	**GEN + AMP (3), TEC + CTX + MEM (7)**	**29**
**20**	**29 + 3/7**	**880**	**GEN + AMP (3), TEC + CTX+MEM (7)**	**30**

* Abbreviations: GEN, gentamicin; AMP, ampicillin; AK, amikacin; CTX, cefotaxime; LEV, levofloxacin; MEM, meropenem; P., penicillin G; SAM, sulbactam-ampicillin; SXT, trimethoprim-sulfamethoxazole; TEC, teicoplanin; TZP, tazobactam-piperacilin; V, vancomycin. ** The underlined and bold represent twin babies.

## Data Availability

The data that support the findings of this study are available from the corresponding author, [AA], upon reasonable request.

## Supplementary Materials

The following supporting information can be downloaded at: https://www.mdpi.com/article/10.3390/antibiotics13100977/s1, File S1: Bray_dist_Phylum; File S2: Jaccard_dist_Phylum; File S3: Phylum All Samples; File S4: Species All Samples; File S5: Bray-Curtis-Species; File S6: Jaccard_dist_Species.
